# Digital health: a tool for mitigating health workforce brain drain in Africa

**DOI:** 10.11604/pamj.2023.45.172.39670

**Published:** 2023-08-21

**Authors:** Oluwaseun Omolade Adekoya, Adeniyi Ayinde Abdulwahab, Greatman Adiela Owhor, Ebere Angela Okoli, Deborah Oluwaseun Shomuyiwa, Onah Ifebuche Maureen, Naomi Chinyere Chikezie

**Affiliations:** 1 Olabisi Onabanjo University, Sagamu, Ogun State, Nigeria,; 2 Faculty of Pharmaceutical Sciences, Bayero University, Kano, Nigeria,; 3 Faculty of Pharmaceutical Sciences, University of Port Harcourt, Choba, Nigeria,; 4 Kaplan Business School, Sydney, Australia,; 5 Faculty of Pharmacy, University of Lagos, Lagos, Nigeria,; 6 Faculty of Pharmaceutical Sciences, University of Nigeria, Nsukka, Enugu, Nigeria

**Keywords:** Digital health, health workforce migration, Africa

## To the editors of the Pan African Medical Journal

Brain drain in the health sector is the migration of skilled health workers from their countries of origin in search of a better standard of living, improved working conditions, access to advanced technology, and stable political conditions [1]. The availability of a sufficient health workforce is critical to optimal healthcare in any region. The World Health Organization recommended a minimum health worker density of 4.45 doctors, nurses, and midwives per 1000 population to ensure the achievement of Universal Health Coverage (UHC) and the Sustainable Development Goals (SDGs) targets [2]. However, a subsequent WHO report estimates that the African region has an average density of 1.55 skilled health workers per 1000 population, which correlates with poor health outcomes and a high disease burden [3].

Several internal and external factors, including better remuneration, quality of life, socioeconomic and political stability in destination countries, poor working conditions, and a high disease burden, have contributed to an increasing trend of health workers migrating from Africa to high-income countries [4]. The African continent's already stressed health systems face additional challenges due to a shortage of skilled health workers caused by these migratory patterns. It can be addressed by adopting digital health strategies.

Digital health (or electronic health) refers to the cost-effective and secure use of ICTs in health and health-related fields, including healthcare services, health surveillance, health education, knowledge, and research [5]. In recent years, digital health has gained much traction as an innovative engine to address the shortage in health delivery services and accelerate the attainment of UHC. Potential benefits include improved access to patient records (electronic health records), provision of quality and remote health services (telemedicine), provision of health information and services via mobile technology (mobile health), and improved knowledge and access to health information for health workers (e-learning), all of which lead to increased healthcare workforce productivity [6]. Telemedicine can alleviate the challenges of limited healthcare workers and long hours spent on routine health cases in hospitals promoting efficiency and reducing the high work burden on healthcare workers. Telehealth addresses healthcare delivery through a mobile application and other technological devices utilized by clinicians and patients, reducing regular physical contact that could otherwise increase health risks [7]. Mobile technology has become a platform for improving medical data and service delivery. By the end of 2018, more than two-thirds of the global population had subscribed to a mobile service, with the African region accounting for 39% of all SIM connections and expected to account for 66% by 2025. The use of mobile technology has increased access to reliable health information, which has improved outcomes for tuberculosis, HIV, and psychiatric disorders, among other conditions [8]. The “Internet of Things (IoT)” is an interconnected device that sends and receives data automatically. In a hospital setting or underserved and remote areas, IoT uses connected medical devices with combined health applications for accelerated diagnosis, easy tracking, and monitoring.

Several African countries have begun to adopt digital health as a strategy to improve health service delivery in patient management, disease surveillance, and prevention. For example, mobile phones have been used in South Africa to access 24/7 communication with a doctor (HelloDoctor) and to verify the authenticity of pharmaceutical products in Ghana (mPedigree). Uganda's public health system used mTrac to report, verify, and analyze data and communicate with its workforce. The SMS for life program, a collaboration between the pharmaceutical company Norvatis and eight African countries, has reduced medicine shortages in primary healthcare facilities by tracking stock levels of essential drugs using mobile phones. Matibubu is a malaria diagnostic device developed and currently in use in Uganda. Cameroon also employs CardioPad, a heart-scanning device that transmits data from rural patients to urban cardiologists for evaluation [9].

Drone technologies are increasingly used in the African region to address the issue of delayed health service delivery to rural and remote communities. They are used to deliver blood and medical supplies faster to patients in critical emergencies. They are also effective in home-based care for elderly and bedridden patients who may experience difficulties traveling long distances. Through a contract with Zipline in 2016, Rwanda became the first African country to utilize drone technology in healthcare delivery, using its aerial vehicles to deliver blood transfusions to remote areas. Lifebank in Nigeria also uses artificial intelligence (AI) and Blockchain technology to supply blood and medical supplies [9,10].

## Conclusion

Despite significant progress in digital innovation in Africa, several factors, such as low digital literacy skills and a lack of connectivity, must still be addressed to increase digital health activity and harness more opportunities for innovation [10]. Several digital solutions are also stand-alone and compete with each other without any system for integration. It creates significant gaps in collecting and analyzing real-time data. These stand-alone solutions cause increased misdiagnosis, inappropriate dispensing, and duplicate services, which impact the overall quality of service delivery [8]. It is essential to interconnect several digital solutions to maximize the potential of digital health in mitigating the health workforce shortage in Africa.
